# Atopic Dermatitis (AD) Related Cord Blood DNA Methylation Patterns Are Linked to Maternal AD

**DOI:** 10.1111/all.70130

**Published:** 2025-11-04

**Authors:** Laura Matzner, Johanna Denkena, Marey Messingschlager, Anke Seegebarth, Rosa Engelhardt, Zhuoxin Peng, Parastoo Kheiroddin, Sebastian D. Mackowiak, Hermann Brenner, Dietrich Rothenbacher, Naveed Ishaque, Michael Kabesch, Roland Eils, Jon Genuneit, Saskia Trump, Irina Lehmann

**Affiliations:** ^1^ Molecular Epidemiology Unit Berlin Institute of Health at Charité – Universitätsmedizin Berlin Berlin Germany; ^2^ Center of Digital Health Berlin Institute of Health (BIH) at Charité – Universitätsmedizin Berlin Berlin Germany; ^3^ Pediatric Epidemiology, Department of Pediatrics, Medical Faculty Leipzig University Leipzig Germany; ^4^ University Children's Hospital Regensburg (KUNO‐Clinics) University of Regensburg, Clinic St. Hedwig Regensburg Germany; ^5^ Division of Clinical Epidemiology and Aging Research German Cancer Research Center (DKFZ) Heidelberg Germany; ^6^ Institute of Epidemiology and Medical Biometry Ulm University Ulm Germany; ^7^ German Center of Child and Adolescent Health (DZKJ) Partner Site Ulm Germany; ^8^ Member of the Research and Development Campus Regensburg (WECARE) at the Clinic St. Hedwig Regensburg Germany; ^9^ German Center of Child and Adolescent Health (DZKJ) Partner Site Berlin Germany; ^10^ German Center for Lung Research (DZL) Associated Partner Site Berlin Germany; ^11^ Intelligent Medicine Institute Fudan University Shanghai China; ^12^ German Center of Child and Adolescent Health (DZKJ) Partner Site Leipzig Germany

**Keywords:** atopic dermatitis, epidemiology, epigenetics, pediatrics

To the Editor,

Atopic dermatitis (AD) is a common chronic inflammatory skin disease that typically begins in early infancy and increases the risk of developing subsequent allergic or immune‐mediated inflammatory diseases. The clinical presentation of AD is highly heterogeneous and influenced by genetic and environmental factors that contribute to a predisposition for AD.

There is evidence from some pioneering studies that epigenetic modifications, in particular DNA methylation changes, play a role in AD‐related pathomechanisms [1, 2]. To investigate the potential impact of epigenetics on early AD development, this study focused on DNA methylation patterns in the cord blood of children who developed AD in their first two years of life, compared to AD‐free controls using a methylome‐wide sequencing approach.

We applied untargeted whole‐genome methylome sequencing (WGMS) as previously described [3] for 62 cord blood samples (*n* = 32 AD, *n* = 30 healthy controls; discovery cohort) from two German birth cohort studies, KUNO‐Kids [4] and the Ulm SPATZ Health Study [5] (Figure 1A). The control group with no known allergic disease background in mothers and child differed from the AD group in terms of known risk factors for AD development, including maternal history of AD (Table S1). To increase reliability, differentially methylated regions (DMRs) were identified with two algorithms, DSS (Dispersion Shrinkage for Sequencing) and metilene, and only overlapping DMRs were considered (Supporting Information). Covariates (child's sex, maternal education, birth weight, number of older siblings, and cold/fever of the mother during pregnancy) used for DSS were identified by principal component analysis (PCA) of the 100,000 most variably methylated CpGs (Figure S1). We identified 110 DMRs (Table S2), with most of them (90%) showing lower DNA methylation in the AD group. DMRs mainly overlapped with enhancer regions (32.7%), and only 5 DMRs were associated with the genotype (gDMRs) (Figure 1B,C). We validated DMRs identified in the discovery cohort using targeted methylation sequencing (TMS) in 977 independent cases from SPATZ and UBCS (Ulm Birth Cohort Study), defining controls more broadly as children without a reported diagnosis of AD, implicating that children with other atopic diseases such as food allergy, asthma, and rhinitis are among the controls [4]. After filtering for metadata completeness and sequencing quality (Table S3), the remaining validation cohort consisted of 756 samples, including 146 children with early‐onset AD (Figure 1D). Logistic regression analysis adjusted for key covariates identified five of the initial 110 DMRs as significantly associated with AD, targeting the genes *FAM120B*, *FAM83H*, *PRUNE2*, *PMAIP1*, and *C20orf197* (Figures 1E and S2). The largest and most consistently AD‐associated DMR across all cohorts and sequencing methods was annotated to *FAM120B*. This region was hypomethylated in WGMS (Δ_methylation_ 4.23%) and TMS (2.47%) in the discovery cohort, and in TMS (1.96%) in the validation cohort (Figure 2A), with 24 of initially 27 CpGs being validated (Figure 1E).

**FIGURE 1:**
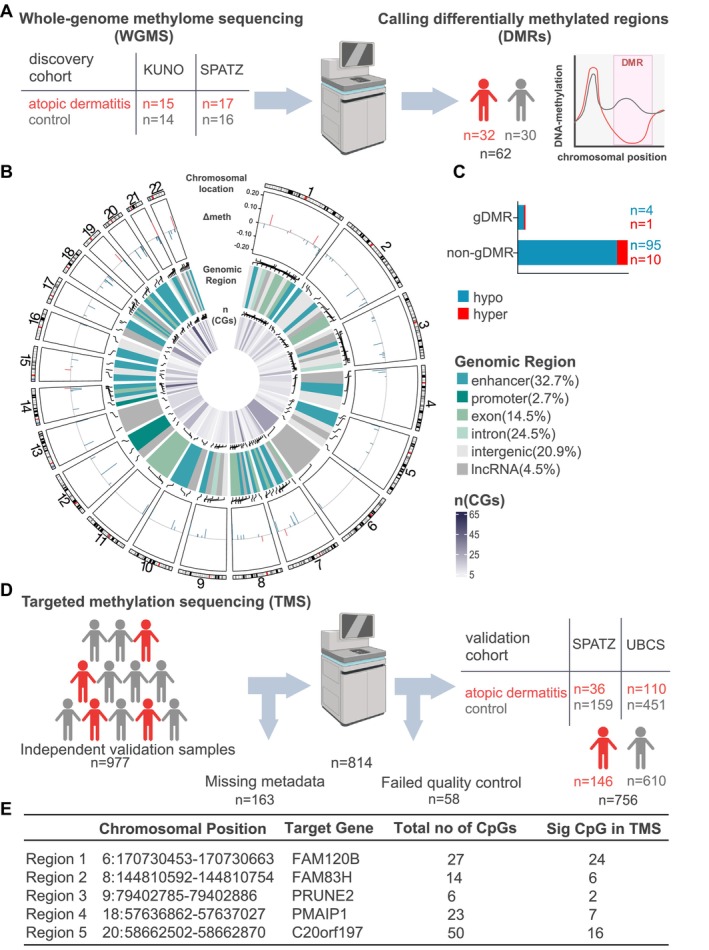
(A) Overview of samples included in the discovery cohort applied for whole‐genome methylome sequencing (WGMS). (B) Circular representation of the distribution of hyper‐ and hypomethylated differentially methylated regions (DMRs) across the genome. Line height within the outer track indicates mean methylation difference. Middle track shows localization of DMRs in different genomic regions, while inner track shows CpG content per DMR. (C) DMRs grouped by genotype association (gDMR)/no genotype association (non‐gDMR) and methylation change direction. (D) Sample overview of the validation cohort analyzed by targeted sequencing (TMS). (E) Table of DMRs still significantly associated with AD after validation. CpG sites within each region exhibited *p*‐values below 0.05.

**FIGURE 2:**
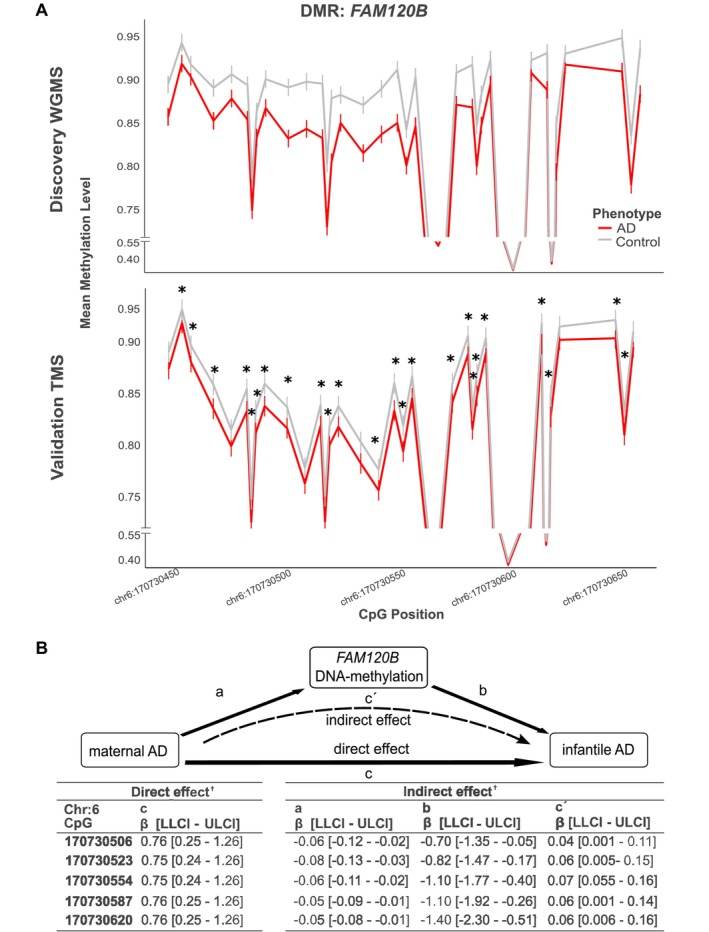
(A) Methylation profiles of hypomethylated FAM120B DMR 161 in discovery cohort with whole‐genome methylome sequencing (WGMS) and validation cohort with targeted methylation sequencing (TMS). Each line chart shows mean methylation values and standard errors per CpG site for the DMRs. Significant differences between atopic dermatitis (AD) and controls were calculated with adjusted logistic regression (*p < 0.05). (B) Analysis of the effect of maternal AD on children's AD risk mediated by FAM120B DMR DNA methylation adjusted for key covariates. c, direct effect; c′, indirect effect; LLCI, lower limit of confidence interval; ULCI, upper limit of confidence interval. ^†^If the confidence interval does not include zero, the variable is significant. Since the dependent variable is dichotomous, *p*‐values are not available.

It is important to note that both in the AD and the control groups other atopic diseases occurred with an expected higher frequency in the AD group. We explicitly tested potential associations between the identified AD‐specific methylation pattern and other atopic diseases such as food allergy, asthma and rhinitis. Importantly, the validated methylation changes were not associated with any other atopic outcomes in the children developing AD, supporting the specificity of this methylation pattern for early AD.

To explore factors underlying the observed methylation changes in the FAM120B DMR, linear regression models were applied, including the key covariates together with additional potential risk factors (Table S1). Among these, maternal AD showed the highest number of associated CpGs (*n* = 7), while maternal smoking was associated with four and season of birth with two CpGs within the FAM120B DMR (Table S4). Therefore, in total, 11 CpGs showed an association with the investigated risk factors, while for the remaining 13 CpGs, no impact of the investigated factors was observed (Table S4), suggesting that additional, yet unidentified, factors may contribute to the observed methylation changes in the FAM120B DMR.

Using the same key covariates as in the WGMS‐based analysis, together with all other identified risk factors associated with FAM120B DMR methylation in a logistic regression analysis, only maternal AD (OR = 2.25, 95% CI: 1.23–4.11) was significantly associated with early AD in the child. Adjusted mediation analysis further indicated that maternal AD, besides a direct impact, may additionally contribute to the child's AD risk through changes in methylation of the FAM120B DMR (Figure 2B), identifying 5 CpGs as potential mediators between maternal and early AD in the child. In contrast, neither maternal smoking nor season of birth showed a significant direct effect on the child's AD risk but contributed indirectly via changes in DNA methylation of the FAM120B DMR (Table S5).

FAM120B is a co‐activator of peroxisome proliferator‐activated receptor gamma (PPARγ), a key regulator of inflammation and immune responses. Notably, PPARγ has been implicated in AD pathophysiology: PPARγ mRNA expression was reportedly increased in monocytes and T‐lymphocytes of AD patients, along with elevated PPARγ protein levels in skin lesions compared to healthy controls [6]. Further, the AD‐related pro‐inflammatory cytokine milieu enhanced PPARγ1 expression [6]. It could be hypothesized that pro‐inflammatory cytokines present in maternal AD may cross the placenta and impact epigenetic programming of fetal immune cells.

Although further, preferably longitudinal, studies are needed to validate a causal relationship mediated by DNA methylation changes, this study provides initial evidence that maternal AD, in addition to other direct or indirect effects, may influence the child's AD susceptibility via epigenetic modifications during the fetal period.

## Author Contributions

I.L., L.M., and S.T. provided project leadership. H.B., D.R., P.K., M.K., J.G. were involved in the recruitment and fieldwork of the cohorts. M.M. performed the whole genome methylation analyses. L.M., R.E., and A.S. performed or guided targeted methylation analyses. L.M., J.D., S.D.M., Z.P., N.I., R.E., S.T., and I.L. supported, performed, or supervised data analysis. L.M., J.D., S.T., and I.L. wrote the initial manuscript. All authors contributed to the final manuscript.

## Conflicts of Interest

The authors declare no conflicts of interest.

## Supporting information


**Table S1:** Characteristics of discovery and validation cohorts grouped by phenotype.
**Figure S1:** PCA of 100.000 most variable CpGs. Association of principal components with potential confounding variables. Heatmaps illustrate the first six principal components (PC1–PC6). Left panel: Spearman's correlation coefficients between continuous features and each PC. Middle panel: Differences in medians for categorical variables with two possible outcomes across the levels of each feature. Right panel: Proportion of variance explained by each categorical variable with three outcomes represented by the Kruskal‐Wallis effect size *η* ^2^ in relation to the PC. Color intensity reflects the magnitude and direction of the association (blue for negative, red for positive), with black asterisks indicating statistically significant associations (adjusted *p* < 0.05).
**Table S2:** Localization and mean methylation of *n* = 110 atopic dermatitis‐related differentially methylated regions (DMRs). Chromosomal positions are displayed as chr:start‐end.
**Table S3:** Mean sequencing quality is grouped by whole‐genome methylome sequencing (WGMS) or targeted methylation sequencing (TMS). Max, maximum; Min, minimum; SD, standard Deviation.
**Figure S2:** Methylation profiles of hypomethylated DMRs in discovery cohort with WGMS and TMS and validation cohort with TMS. Each line chart shows mean methylation values and standard errors per CpG site for the DMRs. Represented in red is the AD group, represented in gray is the corresponding control group. Sig CpGs after validation are indicated by stars. DMR, differentially methylated region.
**Table S4:** Risk factors associated with CpGs in the FAM120B DMR. Risk factors are shown in the top row, with the associated CpG sites listed in the first column. Results of linear regressions are given (standardized b*, [95% LLCI ‐ULCI]).
**Table S5:** Analysis of the effect of risk factors besides maternal AD on children's AD mediated by FAM120B DNA methylation adjusted for key covariates. c, direct effect; c′, indirect effect; LLCI, lower limit of confidence interval; ULCI, upper limit of confidence interval.

## Data Availability

The data that support the findings of this study are available upon request from the corresponding author. The data are not publicly available due to privacy or ethical restrictions.
