# Supplementary material for: Atopic Dermatitis (AD) Related Cord Blood DNA Methylation Patterns Are Linked to Maternal AD
Source: Allergy. 2025 Nov 4;81(2):601–5. doi: 10.1111/all.70130 (PMC12862528; doi:10.1111/all.70130)
Supplement: Supplementary file 1 — Table S1: Characteristics of discovery and validation cohorts grouped by phenotype. Figure S1: PCA of 100.000 most variable CpGs. Association of principal components with potential confounding variables. Heatmaps illustrate the first six principal components (PC1–PC6). Left panel: Spearman's correlation coefficients between continuous features and each PC. Middle panel: Differences in medians for categorical variables with two possible outcomes across the levels of each feature. Right panel: Proportion of variance explained by each categorical variable with three outcomes represented by the Kruskal‐Wallis effect size η 2 in relation to the PC. Color intensity reflects the magnitude and direction of the association (blue for negative, red for positive), with black asterisks indicating statistically significant associations (adjusted p < 0.05). Table S2: Localization and mean methylation of n = 110 atopic dermatitis‐related differentially methylated regions (DMRs). Chromosomal positions are displayed as chr:start‐end. Table S3: Mean sequencing quality is grouped by whole‐genome methylome sequencing (WGMS) or targeted methylation sequencing (TMS). Max, maximum; Min, minimum; SD, standard Deviation. Figure S2: Methylation profiles of hypomethylated DMRs in discovery cohort with WGMS and TMS and validation cohort with TMS. Each line chart shows mean methylation values and standard errors per CpG site for the DMRs. Represented in red is the AD group, represented in gray is the corresponding control group. Sig CpGs after validation are indicated by stars. DMR, differentially methylated region. Table S4: Risk factors associated with CpGs in the FAM120B DMR. Risk factors are shown in the top row, with the associated CpG sites listed in the first column. Results of linear regressions are given (standardized b*, [95% LLCI ‐ULCI]). Table S5: Analysis of the effect of risk factors besides maternal AD on children's AD mediated by FAM120B DNA methylation adjusted for key covariate [file ALL-81-601-s001.docx]

SUPPORTING INFORMATION for

Cord blood DNA methylation patterns associated with later atopic dermatitis are linked to maternal history of AD

Matzner et al.

**Detailed method description**

**Study characteristics and Cohorts**

This study comprises data from three different prospective birth cohort studies: the KUNO-Kids health study, the Ulm SPATZ Health Study and the Ulm Birth Cohort Study (UBCS). KUNO-Kids has been approved by the Ethics Committee of the University of Regensburg (no. 14-101-0347). All participating parents provided written informed consent. SPATZ has been approved by the Ethics Board of Ulm University (no. 311/11). UBCS was approved by the ethics board of Ulm University (no. 98/2000).

**Sample collection of discovery cohort**

Cases and control subjects were evenly distributed across both cohorts. Early onset atopic dermatitis was defined on concurrent parent and pediatrician-reported diagnosis until the age of two years. A total of 32 children with an atopic dermatitis diagnosis and 30 controls were selected for analysis. Controls had no diagnosis or symptoms of atopic dermatitis, atopic rhinitis, food allergy and asthma up to the age 8 years, as well as non-atopic mothers. Cases were included if they met the criteria of the defined outcome parameter and possessed a sufficient quantitative and qualitative amount of genomic DNA. Further information can be found in Table S1.

**Sample collection of validation cohort**

Early onset atopic dermatitis was defined on pediatrician-reported diagnosis until the age of two years. A total of 146 children with an atopic dermatitis diagnosis and 610 controls were selected for analysis. Controls had no diagnosis or symptoms of atopic dermatitis up to the age 6 years. Cases were included if they met the criteria of the defined outcome parameter and possessed a sufficient quantitative and qualitative amount of genomic DNA. Further information can be found in Table S1.

**Whole Genome Methylome Sequencing**

Whole genome methylome sequencing was performed as described by Messingschlager et al [1].

**Gene annotation and definition of enhancer and promoter DMRs**

Genes, enhancers and promoters were annotated as described by Messingschlager et al [1].

**Targeted Methylation Sequencing**

For targeted methylation analysis, 200ng of DNA from umbilical cord blood was used in batches of 16 randomly selected samples. DNA was sheared to an average length of 240–290 bp, with a final library length of 350-450 bp, using the ME220 FocusedUltrasonicator (Covaris). Sequencing libraries were prepared using the NEBNext® EM-seq^TM^ Kit for Twist Bioscience Targeted Methylation Sequencing (New England Biolabs, E7120, distributed by Twist Bioscience). Targeted enrichment was performed using a custom methylation panel and Twist Bioscience protocol for Targeted Methylation Sequencing (REV4, Twist Bioscience). Libraries were sequenced in pools of 96 samples with the NovaSeq 6000 Sequencing System (Illumina) on S4 flow cells.

**Preprocessing of Whole Genome Methylome Sequencing Data**

Preprocessing of whole genome methylome sequencing data was performed as described by Messingschlager et al [1]. The DKFZ AlignmentAndQC workflow was run via OTP to preprocess the TMS data for all samples (Reisinger et al. [3]), including v0.7.8 of bwa (Li and Durbin. [[4]](https://www.zotero.org/google-docs/?t9qnFY)) and v1.0.0 of methylCtools (Hovestadt et al. 2014 [5]). Methylated and unmethylated reads of forward and backward strand were summed up per CpG.

**Principal Component Analysis**

A principal component analysis (PCA) was performed on the methylation levels of 100.000 CpGs with the highest coefficient of variation across all 62 samples on chromosomes 1-22. This was done using python (v3.9.7) with the packages SciPy (v1.7.3) and sklearn (v1.0.2). The top 6 principal components were correlated with cohort variables relating to the newborn’s as well as the mother’s health and lifestyle to assess their association with DNA methylation. The significance of this association was determined using the spearman correlation and the Kruskal-Wallis test for numerical and categorical variables, respectively. Cohort variables with a significant correlation to methylation levels were required to fall under a false discovery rate of 0.05.

**Identification of Differentially Methylated Regions**

Identification of differentially methylated regions (DMRs) was performed as described by Messingschlager et al [1]. DMRs between early atopic dermatitis samples (n=32) and supernormal samples (n=30) were called on chromosomes 1-22. To assess statistical significance, DSS (v2.38.0) applied a Wald test at the single-CpG level with a p-value threshold of 0.01, including adjustment for confounding variables (sex, maternal education, birth weight, number of older siblings and cold of the mother during pregnancy) (Wu et al. [2]). Metilene applied a two-dimensional Kolmogorov-Smirnov (2D-KS) test to DMRs and controlled for multiple testing using Bonferroni correction, with a q-value threshold of 0.01 (Jühling et al. [3]). Only DMRs that met these significance criteria and were detected by both methods (DSS and metilene) were retained for downstream analysis.

**Preprocessing of Targeted Methylome Sequencing Data**

The DKFZ AlignmentAndQC workflow was run via OTP to preprocess the TMS data for all samples (Reisinger et al. [4]), including v0.7.8 of bwa (Li and Durbin. [[5]](https://www.zotero.org/google-docs/?t9qnFY)) and v1.0.0 of methylCtools (Hovestadt et al. 2014 [6]). Methylated and unmethylated reads of forward and backward strand were summed up per CpG.

**Targeted analysis using Regression and Mediator Model**

Logistic regression models were calculated using R v4.1 R (Core Team (2023), _R: A Language and Environment for Statistical Computing _R Foundation for Statistical Computing, Vienna, Austria). The child's sex, maternal education, birth weight, number of older siblings and cold/fever of the mother during pregnancy were introduced as confounding factors in all models. Confounder-adjusted mediation analyses were performed using the PROCESS macro version v3.4.1 for SPSS, Statistical significance of the indirect effect was determined by bootstrapping as implemented in the PROCESS macro. Bias-corrected 90% confidence intervals were derived from the distribution of bootstrap estimates of the indirect effect from random resampling of 5000 samples. Effect sizes of regression analyses are provided as standardized β.

**Supplementary results section**

**Table S1:** Characteristics of discovery and validation cohorts grouped by phenotype.

|  | **Discovery Cohort (n=62)** | | | **Validation Cohort (n=756)** | | |
| --- | --- | --- | --- | --- | --- | --- |
|  | atopic dermatitis (n=32) | control (n=30) | p-value | atopic dermatitis (n=146) | control (n=610) | p-value |
| Child´s Sex, n (%) |  |  |  |  |  |  |
| female | 9 (28.1) | 13 (43.3) | 0.325^†^ | 67 (45.9) | 314 (51.5) | 0.263^†^ |
| male | 23(71.9) | 17 (56.6) |  | 79 (54.1) | 296 (48.5) |  |
| Number of Older Siblings |  |  |  |  |  |  |
| median | 0 | 1 | 0.047^‡^ | 0 | 1 | 0.681^‡^ |
| LQ/UQ | (0 - 1) | (0 - 1) |  | (0 - 1) | (0 - 1) |  |
| Maternal Education |  |  |  |  |  |  |
| low | 2 (6.2) | 1(3.3) | 0.841^†^ | 16 (11) | 64 (10.5) | 0.832^†^ |
| middle | 7 (21.9) | 6 (20) |  | 53 (36.3) | 238(39) |  |
| high | 23 (71.9) | 23 (76.6) |  | 77 (52.7) | 308 (50.5) |  |
| Paternal Education |  |  |  |  |  |  |
| low | 2 (7,4)^¶^ | 2 (8)^¶^ |  | 24 (16,43) | 106 (17,38) | 0.755^†^ |
| middle | 5 (18,5)^¶^ | 1 (4)¶ | 0.2607† | 41 (28,1) | 153 (25,08) |  |
| high | 20 (74,1^¶^ | 22 (88)^¶^ |  | 81 (55,47) | 351 (57,54) |  |
| Birth Weight [g] |  |  |  |  |  |  |
| median | 3700 | 3390 | 0.031^‡^ | 3370 | 3400 | 0.755^‡^ |
| LQ/UQ | (3441- 4106) | (2985-3826) |  | (3075 –3698) | (3120 - 3730) |  |
| Maternal Smoking before Pregnancy |  |  |  |  |  |  |
| yes | 16^¶^ (51.61) | 11 (36.67) | 0.359^†^ | 63 (43.15) | 268 (43.93) | 0.937^†^ |
| no | 15^¶^ (48.39) | 19 (63.33) |  | 83 (56.84) | 342 (56.07) |  |
| Maternal History of Atopic Dermatitis |  |  |  |  |  |  |
| yes | 6 (18.7) | 0 | 0.039^†^ | 18 (12.3) | 36 (5.9) | 0.017^†^ |
| no | 26 (81.3) | 30 (100) |  | 128 (87.7) | 574 (94.1) |  |
| Paternal History of Atopic Dermatitis |  |  |  |  |  |  |
| yes | 3 (21.4)^¶^ | ^¶^ | 0.1727† | 7 (4.8) | 33 (5.4) | 0.9263^†^ |
| no | 11 (78.6)^¶^ | 11 (100)^¶^ |  | 139 (95.2) | 577 (94.6) |  |
| Maternal Age at Birth |  |  |  |  |  |  |
| median | 31 | 33 | 0.079^‡^ | 32.65 | 32.4 | 0.901^‡^ |
| LQ/UQ | (29 - 34) | (32 - 34.7) |  | (29.2 - 35.9) | (29.7 - 35.6) |  |
| Fever/Cold^§^ during Pregnancy |  |  |  |  |  |  |
| yes | 25 (78.1) | 17 (56.7) | 0.125^†^ | 28 (19.2) | 110 (18) | 0.840^†^ |
| no | 7 (21.9) | 13 (43.3) |  | 118 (80.8) | 500 (81) |  |
| Delivery Mode |  |  |  |  |  |  |
| vaginal | 19 (59.4) | 22 (75.9) | 0.273^†^ | 116 (79.5) | 513 (84.1) | 0.220^†^ |
| c section | 13 (40.6) | 7 (24.1) |  | 30 (20.5) | 97 (15.9) |  |
| Gestational Week |  |  |  |  |  |  |
| median | 40 | 40 | 0.282^‡^ | 40 | 40 | 0.185^‡^ |
| LQ/UQ | (39 - 42.5) | (39 - 41) |  | (39 - 41) | (39 - 40) |  |
| Birth Season |  |  |  |  |  |  |
| October-March (Winter) | 13(40.6) | 21(70) | 0.039^†^ | 74 (50.7) | 259 (42.5) | 0.088^†^ |
| April-Sept (Summer) | 19 (59.4) | 9 (30) |  | 72 (49.3) | 351 (57.5) |  |

Abbreviations: LQ = lower quartile. UQ = upper quartile

† Pearson's Chi-squared test with Yates' continuity correction.

‡ from Mann–Whitney U-test.

^§^cold is shown for discovery cohort, fever is shown for validation cohort.

^¶^ variable contains missing data.

**
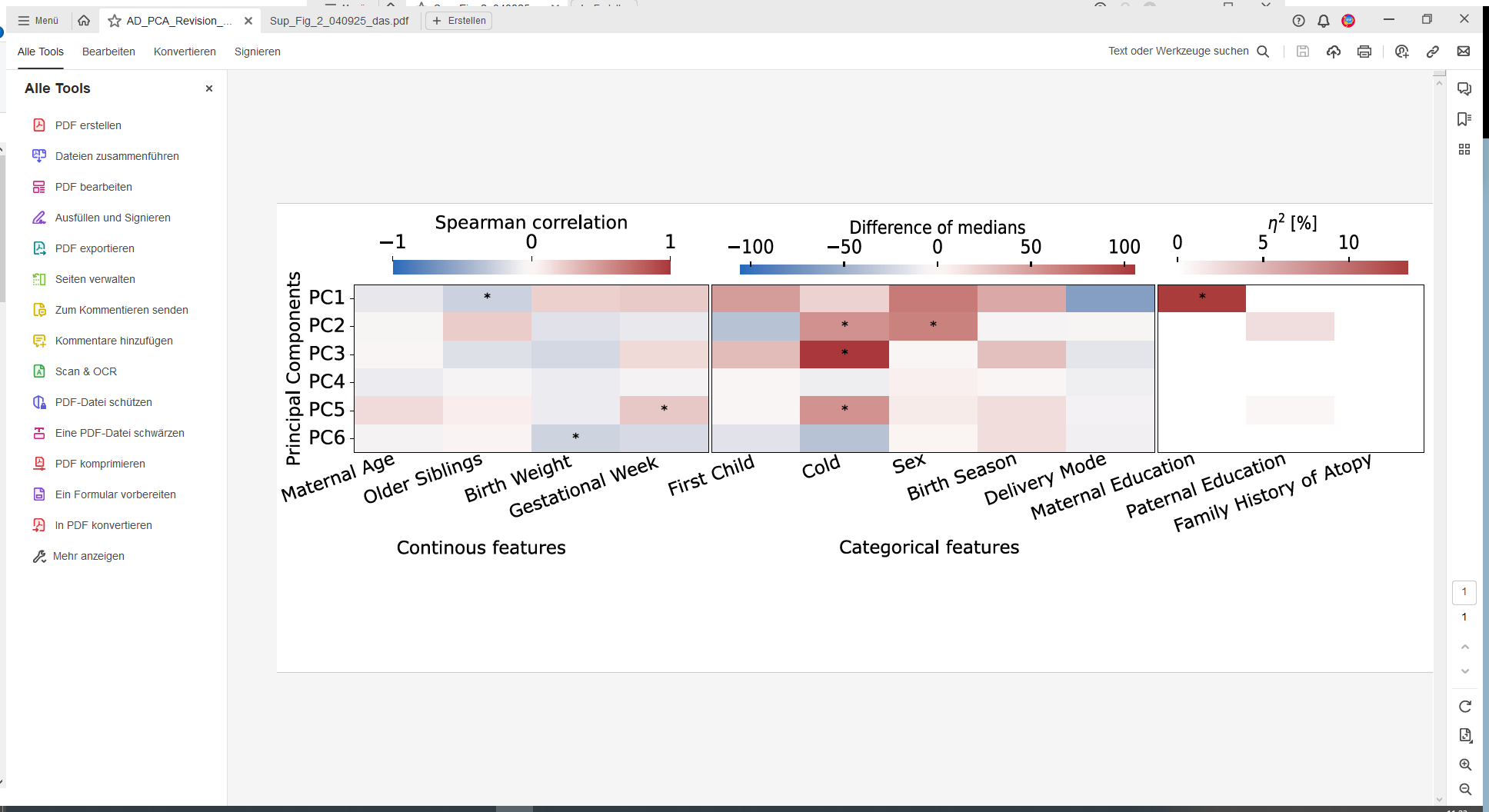
**

**Figure S1**: PCA of 100.000 most variable CpGs. Association of principal components with potential confounding variables. Heatmaps illustrate the first six principal components (PC1–PC6). Left panel: Spearman correlation coefficients between continuous features and each PC. Middle panel: Differences in medians for categorical variables with two possible outcomes across the levels of each feature. Right panel: Proportion of variance explained by each categorical variable with three outcomes represented by the Kruskal-Wallis effect size η² in relation to the PC. Color intensity reflects the magnitude and direction of the association (blue for negative, red for positive), with black asterisks indicating statistically significant associations (adjusted p < 0.05).

**Table S2:** Localization and mean methylation of n=110 atopic dermatitis-related differentially methylated regions (DMRs). Chromosomal positions are displayed as chr:start-end.

| **Differentially Methylated Region** | **Chromosomal Position**  **(chr: start-end)** | **nCpGs** | **DNA-Methylation Difference** | **DNA-Methylation Direction** | **Genomic Localisation** | **Target Genes** | **p-value** | **q-value** |
| --- | --- | --- | --- | --- | --- | --- | --- | --- |
| DMR 1 | 1:113466021-113466138 | 10 | -0.01 | hypo | intron | SLC16A1 | 1.2e-9 | 1.2e-3 |
| DMR 2 | 1:121138153-121138249 | 13 | 0.02 | hyper | enhancer | AL592494.5 | 1.7e-10 | 1.6e-4 |
| DMR 3 | 1:179104099-179104198 | 7 | -0.01 | hypo | intron | TOR3A | 5.6e-12 | 5.5e-6 |
| DMR 4 | 1:193453447-193453525 | 5 | -0.03 | hypo | long non-coding RNA | B3GALT2 | 3.7e-9 | 3.6e-3 |
| DMR 5 | 1:214846635-214846852 | 22 | 0.07 | hyper | intergenic | CENPF | 2.1e-31 | 2.1e-25 |
| DMR 6 | 1:223290407-223290468 | 6 | -0.01 | hypo | intron | TLR5 | 7.1e-9 | 6.9e-3 |
| DMR 7 | 1:228582880-228582949 | 6 | -0.02 | hypo | exon | TRIM11 | 4.5e-9 | 4.4e-3 |
| DMR 8 | 1:23279815-23279956 | 23 | -0.03 | hypo | exon | LACTBL1 | 4.5e-12 | 4.4e-6 |
| DMR 9 | 1:243601074-243601136 | 7 | -0.02 | hypo | intron | SDCCAG8 | 9.5e-9 | 9.2 e-3 |
| DMR 10 | 1:245851731-245851809 | 14 | -0.05 | hypo | exon | KIF26B | 3.7e-21 | 3.6e-15 |
| DMR 11 | 1:43814952-43815078 | 7 | 0.08 | hyper | enhancer | MED8 | 1.5e-11 | 1.5e-5 |
| DMR 12 | 2:10192464-10192516 | 6 | -0.02 | hypo | enhancer | RP11-254F7.3 | 3.7e-10 | 3.6 e-4 |
| DMR 13 | 2:109114736-109114813 | 5 | -0.02 | hypo | intron | LIMS1 | 4.8e-9 | 4.7 e-3 |
| DMR 14 | 2:174081885-174082267 | 17 | -0.10 | hypo | enhancer | CDCA7 | 1.0e-25 | 9.9e-20 |
| DMR 15 | 2:218546167-218546237 | 7 | -0.02 | hypo | intron | DIRC3 | 1.8e-12 | 1.8e-6 |
| DMR 16 | 2:233216600-233216716 | 14 | -0.05 | hypo | intergenic | ALPP | 4.7e-11 | 4.6e-5 |
| DMR 17 | 2:233323867-233323936 | 6 | -0.06 | hypo | exon | ALPI | 1.2e-10 | 1.2 e-4 |
| DMR 18 | 2:233323998-233324054 | 4 | -0.05 | hypo | exon | ALPI | 1.2e-10 | 1.2 e-4 |
| DMR 19 | 2:26594483-26594602 | 12 | -0.02 | hypo | intron | GPR113 | 1.2e-9 | 1.2 e-3 |
| DMR 20 | 2:36872772-36872938 | 12 | -0.02 | hypo | intron | FEZ2 | 5.5e-11 | 5.3e-5 |
| DMR 21 | 3:127056893-127057082 | 20 | -0.06 | hypo | long non-coding RNA | C3orf56 | 7.3e-23 | 5.5e-17 |
| DMR 22 | 3:160757348-160757404 | 4 | -0.01 | hypo | intron | NMD3 | 3.0e-9 | 2.2 e-3 |
| DMR 23 | 3:165976537-165976610 | 10 | -0.02 | hypo | intergenic | BCHE | 3.5e-12 | 2.6e-6 |
| DMR 24 | 3:166395421-166395511 | 5 | -0.04 | hypo | intergenic | ZBBX | 5.4e-11 | 4.1e-5 |
| DMR 25 | 3:171042608-171042663 | 6 | -0.02 | hypo | enhancer | TNIK | 9.2e-9 | 7.0e-3 |
| DMR 26 | 3:178776281-178776361 | 7 | -0.02 | hypo | intron | ZMAT3 | 1.1e-8 | 8.1 e-3 |
| DMR 27 | 3:29089420-29089490 | 8 | -0.02 | hypo | intergenic | RBMS3 | 2.9e-10 | 2.2 e-4 |
| DMR 28 | 3:32763262-32763346 | 5 | -0.02 | hypo | intron | CNOT10 | 1.1e-8 | 8.4 e-3 |
| DMR 29 | 3:41542880-41542938 | 5 | -0.02 | hypo | intron | CTNNB1 | 2.1e-9 | 1.6 e-3 |
| DMR 30 | 4:1522328-1522407 | 11 | -0.10 | hypo | intergenic | NKX1-1 | 5.1e-14 | 3.5e-8 |
| DMR 31 | 4:2366729-2366816 | 7 | -0.05 | hypo | intron | RP11-478C1.7 | 5.4e-12 | 3.8e-6 |
| DMR 32 | 4:25251647-25251747 | 9 | -0.01 | hypo | enhancer | ZCCHC4 | 8.4e-12 | 5.9e-6 |
| DMR 33 | 5:103787728-103787838 | 11 | -0.02 | hypo | intergenic | NUDT12 | 2.3e-9 | 1.6 e-3 |
| DMR 34 | 5:14993383-14993497 | 4 | -0.03 | hypo | enhancer | SEPHS2P1 | 3.6e-10 | 2.5 e-4 |
| DMR 35 | 5:1725245-1725297 | 7 | -0.07 | hypo | enhancer | CTD-2587M23.1 | 2.9e-15 | 2.1e-9 |
| DMR 36 | 5:178548905-178549016 | 6 | -0.04 | hypo | intron | ZNF354C | 3.9e-10 | 2.7 e-4 |
| DMR 37 | 5:68677163-68677233 | 7 | -0.02 | hypo | intron | TAF9 | 9.1e-13 | 6.3e-7 |
| DMR 38 | 6:170730453-170730663 | 27 | -0.04 | hypo | intergenic | FAM120B | 1.6e-10 | 1.1 e-4 |
| DMR 39 | 7:106883675-106883755 | 6 | -0.02 | hypo | intron | HBP1 | 3.4e-10 | 2.3 e-4 |
| DMR 40 | 7:130131142-130131247 | 14 | 0.05 | hyper | enhancer | COPG2 | 6.1e-15 | 4.1e-9 |
| DMR 41 | 7:154684174-154684243 | 4 | -0.07 | hypo | exon | PAXIP1-AS2 | 1.7e-14 | 1.2e-8 |
| DMR 42 | 7:158750653-158750734 | 4 | -0.10 | hypo | enhancer | VIPR2 | 2.3e-12 | 1.5e-6 |
| DMR 43 | 7:6692403-6692546 | 22 | -0.01 | hypo | exon | AC073343.1 | 2.6e-10 | 1.8 e-4 |
| DMR 44 | 7:75779778-75779917 | 16 | -0.09 | hypo | intergenic | SRRM3 | 4.3e-11 | 2.9e-5 |
| DMR 45 | 7:87260766-87260864 | 8 | -0.02 | hypo | intron | RUNDC3B | 1.0e-8 | 7.0 e-3 |
| DMR 46 | 7:90895558-90895630 | 6 | -0.07 | hypo | enhancer | AKAP9 | 2.9e-18 | 2.0e-12 |
| DMR 47 | 7:90895697-90895762 | 7 | -0.07 | hypo | enhancer | AKAP9 | 2.9e-18 | 2.0e-12 |
| DMR 48 | 8:103547244-103547362 | 7 | -0.03 | hypo | enhancer | KB-1980E6.3 | 1.8e-8 | 1.0 e-2 |
| DMR 49 | 8:113095395-113095461 | 7 | -0.03 | hypo | intergenic | na | 4.7e-10 | 2.7 e-4 |
| DMR 50 | 8:125463519-125463575 | 4 | -0.03 | hypo | enhancer | NSMCE2 | 1.5e-11 | 8.8e-6 |
| DMR 51 | 8:144810042-144810101 | 13 | -0.08 | hypo | exon | FAM83H | 4.6e-12 | 2.7e-6 |
| DMR 52 | 8:144810340-144810406 | 14 | -0.10 | hypo | exon | FAM83H | 9.4e-16 | 5.5e-10 |
| DMR 53 | 8:144810592-144810754 | 14 | -0.11 | hypo | exon | FAM83H | 1.2e-20 | 7.2e-15 |
| DMR 54 | 8:1497083-1497160 | 12 | -0.06 | hypo | exon | DLGAP2 | 1.5e-12 | 8.8e-7 |
| DMR 55 | 8:37699593-37699829 | 34 | 0.04 | hyper | enhancer | BRF2 | 1.7e-25 | 9.9e-20 |
| DMR 56 | 8:48958887-48958970 | 7 | -0.02 | hypo | intron | UBE2V2 | 7.0e-11 | 4.1e-5 |
| DMR 57 | 9:105249524-105249589 | 9 | -0.02 | hypo | long non-coding RNA | CYLC2 | 4.6e-9 | 2.3 e-3 |
| DMR 58 | 9:107482635-107483056 | 7 | -0.02 | hypo | intergenic | OR13D1 | 7.3e-9 | 3.8 e-3 |
| DMR 59 | 9:140311750-140311803 | 5 | -0.08 | hypo | enhancer | LCNL1 | 1.2e-35 | 6.4e-30 |
| DMR 60 | 9:79402785-79402886 | 6 | -0.06 | hypo | intron | PRUNE2 | 3.8e-14 | 1.9e-8 |
| DMR 61 | 9:90728177-90728231 | 8 | -0.01 | hypo | intergenic | CDK20 | 7.6e-9 | 3.9 e-3 |
| DMR 62 | 10:134332294-134332443 | 8 | -0.08 | hypo | enhancer | INPP5A | 1.3e-10 | 7.3e-5 |
| DMR 63 | 10:33338456-33338513 | 5 | -0.02 | hypo | intron | ITGB1 | 9.4e-9 | 5.4 e-3 |
| DMR 64 | 10:43846258-43846321 | 7 | -0.07 | hypo | enhancer | FXYD4 | 1.0e-9 | 6.0 e-4 |
| DMR 65 | 11:6291980-6292049 | 5 | -0.07 | hypo | exon | CCKBR | 3.9e-17 | 2.2e-11 |
| DMR 66 | 11:6292204-6292444 | 21 | -0.08 | hypo | exon | CCKBR | 3.9e-17 | 2.2e-11 |
| DMR 67 | 12:12071868-12071967 | 6 | -0.02 | hypo | intergenic | BCL2L14 | 1.6e-8 | 9.3 e-3 |
| DMR 68 | 12:124457754-124457820 | 16 | -0.03 | hypo | promoter | ZNF664 | 2.5e-13 | 1.4e-7 |
| DMR 69 | 12:57083554-57083870 | 14 | -0.14 | hypo | promoter | COQ10A | 6.9e-9 | 4.0 e-3 |
| DMR 70 | 13:112986262-112986542 | 11 | -0.09 | hypo | intergenic | SPACA7 | 2.9e-14 | 1.1e-8 |
| DMR 71 | 13:24914721-24914864 | 11 | -0.07 | hypo | intergenic | AL359736.1 | 5.5e-14 | 2.1e-8 |
| DMR 72 | 14:100141674-100142051 | 46 | -0.08 | hypo | exon | CYP46A1 | 1.6e-17 | 6.2e-12 |
| DMR 73 | 14:102974001-102974067 | 12 | 0.04 | hyper | enhancer | TECPR2 | 8.7e-10 | 3.3 e-4 |
| DMR 74 | 14:24780514-24780692 | 22 | -0.04 | hypo | promoter | PABPN1 | 2.9e-29 | 1.1e-23 |
| DMR 75 | 14:91761739-91761803 | 5 | -0.02 | hypo | intron | GPR68 | 4.1e-9 | 1.6 e-3 |
| DMR 76 | 14:95156527-95156706 | 13 | -0.08 | hypo | enhancer | GSC | 1.3e-8 | 4.8 e-3 |
| DMR 77 | 15:40268719-40268844 | 17 | 0.04 | hyper | enhancer | BAHD1 | 2.3e-9 | 8.5 e-4 |
| DMR 78 | 15:52195002-52195095 | 5 | -0.01 | hypo | intron | MAPK6 | 2.5e-8 | 9.1 e-3 |
| DMR 79 | 15:78186587-78186734 | 17 | -0.05 | hypo | enhancer | CSPG4P13 | 1.1e-15 | 4.0e-10 |
| DMR 80 | 16:1035273-1035327 | 8 | -0.01 | hypo | enhancer | SOX8 | 1.6e-8 | 6.7 e-3 |
| DMR 81 | 16:87700717-87700769 | 4 | -0.05 | hypo | intron | AC010536.1 | 2.2e-8 | 9.4 e-3 |
| DMR 82 | 17:11092052-11092168 | 10 | -0.02 | hypo | intergenic | SHISA6 | 2.5e-9 | 1.1 e-3 |
| DMR 83 | 17:48858638-48858877 | 30 | -0.05 | hypo | enhancer | WFIKKN2 | 3.5e-11 | 1.6e-5 |
| DMR 84 | 18:28685514-28685568 | 4 | -0.02 | hypo | long non-coding RNA | DSC2 | 4.4e-10 | 1.4 e-4 |
| DMR 85 | 18:52663521-52663591 | 7 | -0.02 | hypo | intergenic | CCDC68 | 1.4e-8 | 4.4 e-3 |
| DMR 86 | 18:57636862-57637027 | 23 | -0.07 | hypo | enhancer | PMAIP1 | 2.8e-14 | 8.6e-9 |
| DMR 87 | 18:57637082-57637173 | 9 | -0.06 | hypo | exon | PMAIP1 | 2.8e-14 | 8.6e-9 |
| DMR 88 | 18:59221511-59221716 | 25 | -0.06 | hypo | enhancer | CDH20 | 5.1e-16 | 1.6e-10 |
| DMR 89 | 18:72837484-72837548 | 8 | -0.05 | hypo | intergenic | ZADH2 | 1.0e-10 | 3.1e-5 |
| DMR 90 | 18:76765876-76765947 | 10 | -0.04 | hypo | intergenic | SALL3 | 1.1e-8 | 3.4 e-3 |
| DMR 91 | 19:49071869-49072026 | 10 | 0.07 | hyper | enhancer | SULT2B1 | 8.5e-9 | 3.2 e-3 |
| DMR 92 | 19:57175945-57176011 | 13 | -0.02 | hypo | exon | ZNF835 | 1.5e-10 | 5.6e-5 |
| DMR 93 | 19:57351321-57351387 | 4 | -0.06 | hypo | enhancer | ZNF583 | 6.1e-14 | 2.3e-08 |
| DMR 94 | 20:32255343-32255403 | 11 | -0.07 | hypo | enhancer | CBFA2T2 | 2.6e-28 | 7.7e-23 |
| DMR 95 | 20:55787426-55787525 | 7 | -0.08 | hypo | enhancer | BMP7 | 2.8e-9 | 8.3 e-4 |
| DMR 96 | 20:58662502-58662870 | 50 | -0.03 | hypo | enhancer | C20orf197 | 1.2e-8 | 3.4 e-3 |
| DMR 97 | 21:38267288-38267361 | 5 | -0.02 | hypo | intron | HLCS | 3.1e-10 | 5.0e-5 |
| DMR 98 | 21:40038781-40038839 | 5 | -0.01 | hypo | long non-coding RNA | ERG | 2.6e-8 | 4.2 e-3 |
| DMR 99 | 21:44573599-44573688 | 5 | -0.05 | hypo | enhancer | AP001631.10 | 3.6e-9 | 5.8 e-4 |
| DMR 100 | 21:44573738-44574073 | 30 | -0.06 | hypo | enhancer | AP001631.10 | 3.9e-12 | 6.4e-7 |
| DMR 101 | 21:46975748-46975809 | 6 | -0.04 | hypo | enhancer | AP001469.9 | 5.0e-11 | 7.9e-6 |
| DMR 102 | 21:48119132-48119280 | 21 | 0.11 | hyper | intergenic | PRMT2 | 9.3e-32 | 1.5e-26 |
| DMR 103 | 22:21021972-21022305 | 30 | -0.06 | hypo | intergenic | SERPIND1 | 2.0e-16 | 4.2e-11 |
| DMR 104 | 22:21022365-21022463 | 12 | -0.05 | hypo | intergenic | SERPIND1 | 2.0e-16 | 4.2e-11 |
| DMR 105 | 22:28770699-28770773 | 6 | -0.02 | hypo | intron | TTC28 | 4.8e-9 | 1.0 e-3 |
| DMR 106 | 22:41787770-41787877 | 8 | -0.02 | hypo | intron | TEF | 1.4e-8 | 2.9 e-3 |
| DMR 107 | 22:43498475-43498544 | 4 | -0.02 | hypo | enhancer | BIK | 6.9e-10 | 1.5 e-4 |
| DMR 108 | 22:45832697-45833137 | 33 | 0.10 | hyper | intergenic | RIBC2 | 7.6e-21 | 1.6e-15 |
| DMR 109 | 22:49875585-49875646 | 4 | 0.14 | hyper | intron | C22orf34 | 2.4e-8 | 5.2 e-3 |
| DMR 110 | 22:50981708-50981780 | 7 | -0.02 | hypo | enhancer | TYMP | 4.2e-9 | 8.9 e-4 |

**Table S3:** Mean sequencing quality is grouped by whole-genome methylome sequencing (WGMS) or targeted methylation sequencing (TMS). SD = Standard Deviation, Min = Minimum, Max = Maximum

|  |  | **Mean Genome Coverage** | **Mean % of mapped reads** | **Mean % of duplicate reads** | **Mean % of properly pair reads** |
| --- | --- | --- | --- | --- | --- |
| **WGMS** | Mean  SD  Min/Max | 27.29  27.48  0.17/106.83 | 99.86  0.13  99.55/99.99 | 15.53  7.88  4.83/46.44 | 82.79  11.02  33.67/96.91 |
| **TMS** | Mean  SD  Min/Max | 0.74  0.57  0.01/5.76 | 99.68  0.072  99.39/99.85 | 17.22  9.31  2.95/53.75 | 88.24  3.30  53.82/94.19 |

**
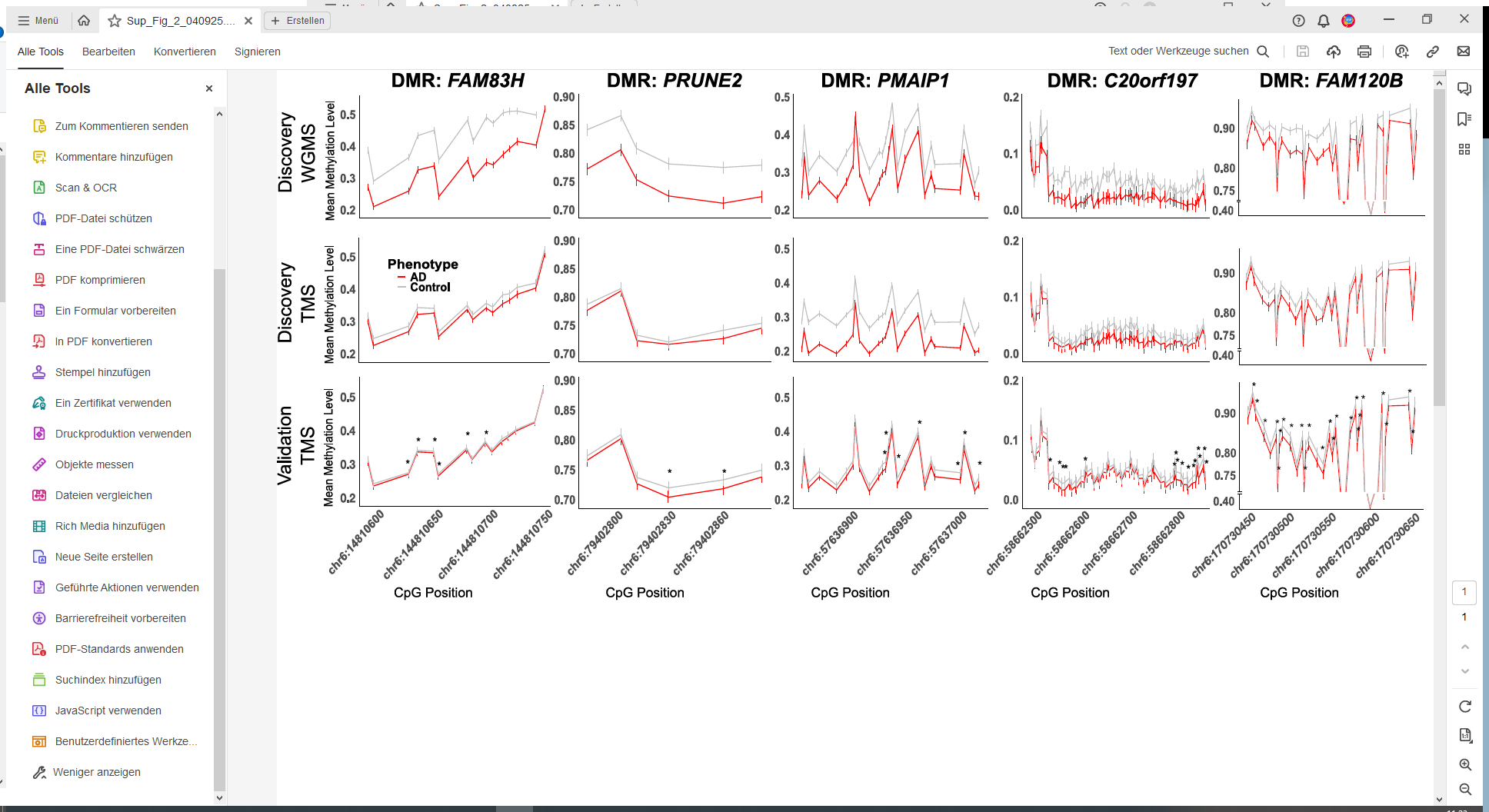
**

**Figure S2:** Methylation profiles of hypomethylated DMRs in discovery cohort with WGMS and TMS and validation cohort with TMS. Each line chart shows mean methylation values and standard errors per CpG site for the DMRs. Represented in red is the AD group, represented in grey is the corresponding control group. Sig CpGs after validation are indicated by stars. DMR = differentially methylated region.

**Table S4:** Risk factors associated with CpGs in the FAM120B DMR. Risk factors are shown in the top row, with the associated CpG sites listed in the first column. Results of linear regressions are given (standardized b*, [95 % LLCI -ULCI]).

| **CpG Position** | **Maternal Atopic Dermatitis**  b*, [LLCI -ULCI] | **Maternal Smoking before Pregnancy**  b*, [LLCI -ULCI] | **Birth Season**  b*, [LLCI -ULCI] |
| --- | --- | --- | --- |
| Chr6: 170730463 |  |  | - 0.07 [-0.15– -0.003] |
| Chr6: 170730506 | -0.08 [-0.15– - 0.006] |  |  |
| Chr6: 170730521 | -0.08 [-0.15– -0.004] |  | -0.07 [-0.15– -0.001] |
| Chr6: 170730523 | -0.09 [-0.16– - 0.019] |  |  |
| Chr6: 170730554 | -0.08 [-0.15– - 0.010] | -0.07 [-0.14– -0.001] |  |
| Chr6: 170730558 |  | -0.08 [-0.15– -0.010] |  |
| Chr6: 170730562 |  | -0.07 [-0.14– -0.001] |  |
| Chr6: 170730580 | -0.07 [-0.14– -0.002] |  |  |
| Chr6: 170730587 | -0.08 [-0.01– -0.005] |  |  |
| Chr6: 170730591 |  | -0.08 [-0.15– -0.006] |  |
| Chr6: 170730620 | -0.08 [-0.15– - 0.008] |  |  |

**Table S5:** Analysis of the effect of risk factors besides maternal AD on children's AD mediated by FAM120B DNA methylation adjusted for key covariates. c´: indirect effect, c: direct effect, LLCI: low level confidence interval, ULCI: upper level confidence interval.

| **Direct effect** | | |  | **Indirect effect**^†^ | | |
| --- | --- | --- | --- | --- | --- | --- |
| **CpG Position** | **c**  **β [LLCI - ULCI]** | |  | **a**  **β [LLCI - ULCI]** | **b**  **β [LLCI - ULCI]** | **c´**  **β [LLCI - ULCI]** |
| **Maternal Smoking before Pregnancy** | | **Maternal Smoking before Pregnancy** | | | | |
| **Chr6:170730554** | -0.06 [-0.37 - 0.25] | |  | -0.03 [-0.05 - -0.01] | -1.18 [-1.87 - -0.50] | 0.35 [0.004- 0.07] |
| **Chr6:170730558** | -0.07 [-0.38 - 0.24] | |  | -0.03 [-0.06 - -0.01] | -1.13 [-1.83- -0.43] | 0.04 [0.007- 0.08] |
| **Chr6:170730562** | -0.06 [-0.37 - 0.25] | |  | -0.03 [-0.05 - -0.01] | -1.19 [-1.94 - -0.43] | 0.03 [0.004 - 0.06] |
| **Chr6:170730591** | -0.06 [-0.38 - 0.25] | |  | -0.03 [-0.05 - -0.01] | -1.28 [-2.10 - -0.49] | 0.04 [0.006 - 0.07] |
| **Birth Season** | | **Birth Season** | | | | |
| **Chr6:170730463** | -0.09 [-0.46 - 0.27] | |  | -0.02 [-0.04 - -0.003] | -1.10 [-1.93 - -0.30] | 0.03 [0.001 - 0.06] |
| **Chr6:170730521** | -0.09 [-0.40 - 0.21] | |  | -0.03 [-0.05 - -0.001] | -0.78 [-1.55 - -0.20] | 0.02 [0.001 - 0.06] |

^†^ If the confidence interval does not include zero, the variable is significant. Since the dependent variable is dichotomous, p-values are not available.

**Data Availability**

Data are available upon reasonable request to the corresponding author.

ADDITIONAL REFERENCES

[1] Messingschlager M. et al. Genome-wide DNA methylation sequencing identifies epigenetic perturbations in the upper airways under long-term exposure to moderate levels of ambient air pollution. Environmental Research, 2023, September; https://doi.org/10.1016/j.envres.2023.116413

[2] Wu H. et al. Detection of differentially methylated regions from whole-genome bisulfite sequencing data without replicates. Nucleic Acids Research, 2015, July; https://doi.org/10.1093/nar/gkv715

[3] Jühling F. et al. metilene: fast and sensitive calling of differentially methylated regions from bisulfite sequencing data. Genome Research, 2016, 26(2):256–262. https://doi.org/10.1101/gr.196394.115

[4] Reisinger E. et al. OTP: An automatized system for managing and processing NGS data. Journal of Biotechnology, 2017, August; https://doi.org/10.1016/j.jbiotec.2017.08.006

[5] Li H. Durbin R. Fast and accurate short read alignment with Burrows–Wheeler transform. Bioinformatics, 2009, May; 25(14):1754–1760. https://doi.org/10.1093/bioinformatics/btp324

[6] Hovestadt V. Jones D.T.W. Picelli S. et al. Decoding the regulatory landscape of medulloblastoma using DNA methylation sequencing. Nature, 2014, May; 510:537–541. https://doi.org/10.1038/nature13268
